# Improving natural ventilation in hospital waiting and consulting rooms to reduce nosocomial tuberculosis transmission risk in a low resource setting

**DOI:** 10.1186/s12879-019-3717-9

**Published:** 2019-01-25

**Authors:** A. Roderick Escombe, Eduardo Ticona, Víctor Chávez-Pérez, Manuel Espinoza, David A. J. Moore

**Affiliations:** 1 Department of Infectious Diseases & Immunity and the Wellcome Trust Centre for Clinical Tropical Medicine, Imperial College London, London, UK; 2 Hospital Nacional Dos de Mayo, Lima, Peru; 3 Universidad Nacional Mayor de San Marcos, Lima, Peru; 4 TB Centre, London School of Hygiene and Tropical Medicine, London, UK; 5 Universidad Peruana Cayetano Heredia, Lima, Peru

**Keywords:** Natural ventilation, Tuberculosis, Tuberculosis transmission, Airborne transmission, Nosocomial transmission, Waiting room

## Abstract

**Background:**

TB transmission in healthcare facilities is an important public health problem, especially in the often-overcrowded settings of HIV treatment scale-up. The problem is compounded by the emergence of drug resistant TB. Natural ventilation is a low-cost environmental control measure for TB infection control where climate permits that is suited to many different areas in healthcare facilities. There are no published data on the effect of simple structural modifications to existing hospital infrastructure to improve natural ventilation and reduce the risk of nosocomial TB transmission.

The purpose of this study was to measure the effect of simple architectural modifications to existing hospital waiting and consulting rooms in a low resource setting on (a) improving natural ventilation and (b) reducing modelled TB transmission risk.

**Methods:**

Room ventilation was measured pre- and post-modification using a carbon dioxide tracer-gas technique in four waiting rooms and two consulting rooms in two hospitals in Lima, Peru. Modifications included additional windows for cross-ventilation (*n* = 2 rooms); removing glass from unopenable windows (*n* = 2); creation of an open skylight (*n* = 1); re-building a waiting-room in the open air (*n* = 1). Changes in TB transmission risk for waiting patients, or healthcare workers in consulting rooms, were estimated using mathematical modelling.

**Results:**

As a result of the infrastructure modifications, room ventilation in the four waiting rooms increased from mean 5.5 to 15; 11 to 16; 10 to 17; and 9 to 66 air-changes/hour respectively; and in the two consulting rooms from mean 3.6 to 17; and 2.7 to 12 air-changes/hour respectively. There was a median 72% reduction (inter-quartile range 51–82%) in calculated TB transmission risk for healthcare workers or waiting patients. The modifications cost <US$75 in four rooms, and US$1000 and US$7000 in the remaining two rooms.

**Conclusions:**

Simple modifications to existing hospital infrastructure considerably increased natural ventilation, and greatly reduced modelled TB transmission risk at little cost.

## Introduction

TB transmission in healthcare facilities is an important public health problem. Occupational TB has been widely documented in the developed world, and is increasingly recognised in low and middle income countries [1, 2]. In addition to staff, patients and visitors to healthcare facilities are at risk of infection. TB transmission in other institutional settings such as prisons, homeless shelters and schools is also important [3–5].

The dual epidemics of HIV infection and drug-resistant TB conspire to compound the problem of institutional TB transmission. HIV patients latently-infected with TB are more likely to develop active TB disease [6], and thus cause onward transmission via the airborne route. Indeed in high TB burden countries, a high proportion of HIV cases themselves present with active TB, forming a significant pool of TB infection within waiting areas of HIV clinics. Furthermore, HIV infection increases hospitalisation and attendance at healthcare facilities. The roll out of HIV care may inadvertently promote the risk of airborne TB transmission through the congregation of highly susceptible patients with a high incidence of TB in settings which are often overcrowded, such as anti-retroviral treatment centres [7]. TB drug resistance may increase overall TB transmission in a number of ways, including diagnostic delay and prolonged periods of treatment. There may be frequent attendances at healthcare facilities whilst drug-resistant TB patients are treated unsuccessfully with first-line anti-TB drugs prior to correct diagnosis. Such inadequately treated patients may be highly infectious [8]. Nosocomial transmission has been strongly implicated in the emergence of extensively drug resistant TB in South Africa in health care settings where airborne infection control was poor or absent [9, 10].

Airborne TB transmission depends on a number of factors, including source strength (for example cough frequency of an undiagnosed TB patient) and the number of susceptible persons exposed [11]. A major determinant of transmission is room ventilation with fresh air, which serves to dilute the concentration of airborne infectious particles. Room ventilation may be provided by mechanical ventilation systems that may also deliver negative pressure. Such systems require specific expertise to design and are expensive to install and maintain, and are inappropriate for many low resource settings where the burden of TB is highest. Natural ventilation by simply opening the windows may provide higher rates of air exchange for little or no cost, but is climate dependant [12]. TB infection control guidelines recommend 6–12 air-changes/hour (ACH) room ventilation for high risk settings [13, 14]. Even if such environmental control measures are implemented, they are often limited to areas considered high risk, such as TB wards. However it is untreated TB patients prior to diagnosis, or inadequately treated drug-resistant TB patients, who are likely to be the most infectious [15]. These patients are commonly found in areas such as emergency rooms, waiting rooms, out-patient clinics, and X-ray departments. These areas are often overcrowded, especially in low resource settings, and are often not the focus of TB infection control efforts.

We studied the effect of simple architectural modifications to existing infrastructure to improve natural ventilation in out-patient consulting rooms and waiting rooms in hospitals in a high TB burden setting, and used mathematical modelling to estimate reductions in the risk of TB transmission to patients and healthcare workers.

## Methods

### Setting

Two out-patient consulting rooms and four waiting rooms in two general hospitals in high TB prevalence areas of Lima, Perú were studied.

### Interventions to improve natural ventilation

**Fig. 1:**
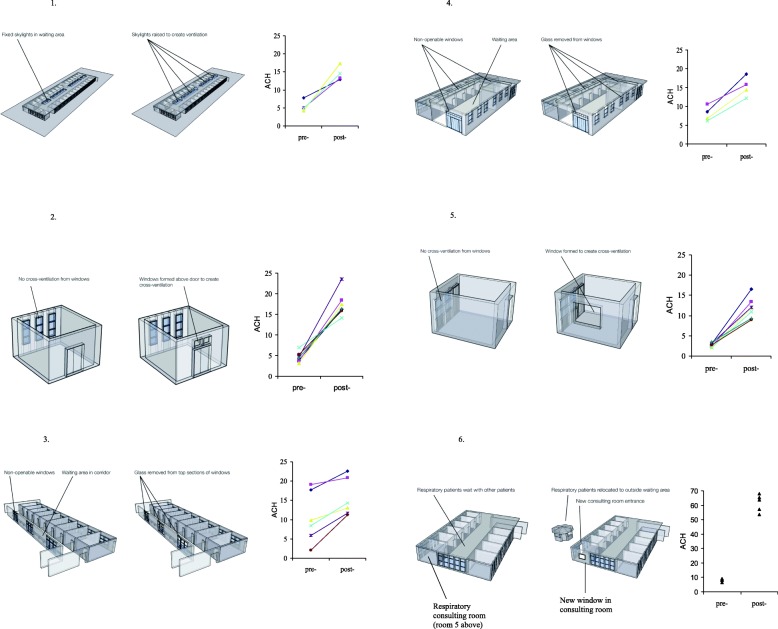
Schematics are shown of the six rooms in the study, with the modification to improve natural ventilation annotated. Room ventilation (air-changes/hour; ACH) is shown alongside as measured pre- and post-modification. The following 6 rooms are shown: 1. General medical out-patients waiting room. 2. General medical out-patients consulting room. 3. X-ray department waiting room. 4. Respiratory medicine & TB clinic waiting room. 5. Respiratory medicine out-patients consulting room. 6. General medical and respiratory medicine out-patients waiting room

Simple modifications were made to 6 rooms, detailed below and shown in Fig. 1. Sequential measurements of room ventilation were made to capture the effect of the intervention. In order to measure pre- and post-intervention ventilation under the same conditions of wind and temperature, plastic sheeting and strong tape were used to seal the new apertures to re-create the pre-intervention configuration.

1. General medical out-patients waiting room: 26 consulting rooms open onto this large hall; large doorways open to the street and two hospital courtyards. The intervention involved raising 4 sections of the sealed glass roof on 1 m stilts to create open skylights, costing approximately USD 1000. Pre-intervention room ventilation was measured with 3 doorways open, new skylights sealed with plastic sheeting. The post-intervention ventilation was measured with 3 doorways open and the skylights open.
2. General medical out-patients consulting room: This room has nine windows facing the street, which open partially; a door leads to the waiting room described above. The intervention was to repair unopenable windows above the door, to permit cross-ventilation, and cost USD 25. Pre-intervention room ventilation was measured with original windows partially open, door closed; post-intervention ventilation was measured as above, but with the new windows above the door open as well. The doctor’s seat was located next to the windows facing the street, and the patient was consulted across a desk.
3. X-ray department waiting room: This busy corridor has doors opening along one side to the X-ray rooms, and had 27 unopenable windows to the outside on the opposite wall, where patients wait on benches. The intervention involved removing glass from 25% of the total window area. Pre-intervention room ventilation was measured with doors open, windows without glass sealed with plastic sheeting; post-intervention ventilation was measured with doors open, and plastic sheeting removed leaving 25% of total window area open.
4. Respiratory medicine out-patients & TB clinic waiting room: This room is shared between respiratory out-patients and the National TB Control Programme. It has a main entrance, and a second door to the outside, where sputum samples are collected. One wall has multiple windows opening to the outside; the three remaining walls had a row of high unopenable windows. The intervention involved removing the glass from the top row of windows on all four walls to facilitate cross ventilation. Pre-intervention room ventilation was measured with 2 doors and 18% of window area open, the maximum openable prior to the intervention (the new apertures created by removing glass were sealed with plastic sheeting). Post-intervention ventilation was measured with 2 doors open, and 70% of window area open (plastic sheeting removed).
5. Respiratory medicine out-patients consulting room: This room, situated on the corner of the building, had a door to the waiting room, a posterior door to the outside, and one window on the posterior wall. Consultations were conducted with the window open and both doors closed. The intervention was to construct a new window in the side wall, to allow cross ventilation. This cost less than USD 100. Pre-intervention room ventilation was measured with the original window open, both doors closed; post-intervention ventilation was measured with both windows open, doors closed. The consulting doctor’s chair was located adjacent to the new window, and the patient was consulted across a desk.
6. General medical and respiratory medicine out-patients waiting room: This room was shared by patients attending respiratory medicine, gastroenterology, cardiology, dermatology, and paediatric out-patient clinics. The intervention involved building a separate waiting room outside specifically for respiratory out-patients, who now used the posterior door to access the respiratory consulting room detailed above. The cost was approximately USD 7000. Pre-intervention ventilation was measured in the original waiting room and post-intervention ventilation in the new waiting room.

### Measurement of ventilation

Room ventilation was measured on 4–7 occasions in each room using a carbon dioxide (CO_2_) tracer-gas technique, as previously described [12, 16]. Briefly, all apertures such as windows, doors or skylights were closed or sealed with plastic sheeting and tape. CO_2_ was released and mixed with room air using fans to achieve near uniform concentration. After 5 min, certain windows and/or doors were opened to achieve the pre-intervention working conditions configuration of room ventilation. After a further 5 min, appropriate additional apertures were opened (e.g. new windows opened, or new apertures unsealed by removing the plastic sheeting) to achieve the post-intervention configuration of ventilation. CO_2_ levels were measured throughout at 1 minute intervals using a centrally located infra-red gas analyser (Gas Data Ltd., Coventry, UK). Air-changes/hour (ACH) were calculated by plotting the natural logarithm of CO_2_ concentration against time separately for the pre-intervention and post-intervention configurations.

### Modelling risk of TB infection

The risk of TB infection for patients waiting in the waiting room, or for healthcare staff working in the consulting rooms, was calculated in each room under pre-intervention and post-intervention conditions of room ventilation, using the Wells-Riley model of airborne infection [11]. This is defined as: C=S(1-e^–Iqpt/Q^) where: C = number of new cases, S = number of susceptibles exposed, I = number of infectious source cases, q = infectious quanta produced per hour by source cases, p = pulmonary ventilation of susceptibles (m^3^/hour)., t = exposure time (hours), and Q = absolute ventilation of room (m^3^/hour).

Data regarding numbers of patients passing through each room or healthcare workers in each clinic room (S = susceptibles), number of untreated smear positive TB cases attended to (I = infectors) and approximate waiting times (t = exposure time), were collected by direct observation, review of clinic records, and staff interview. The term “quantum” (‘q’) is used to describe the “infectious dose” for TB, and a value of 13 was used, that calculated for an untreated case of infectious TB in a well documented office outbreak [17]. Pulmonary ventilation was assumed to be 0.6 m^3^/hour [11]. Absolute ventilation (Q m^3^/h) was calculated for each room by multiplying ACH by room volume (m^3^). The following assumptions were made: infectious TB droplet nuclei were distributed uniformly throughout the air; the probability of encountering an infectious TB patient was uniform across the day; no personal respiratory devices were worn.

Institutional approval was provided by Asociación Benéfica PRISMA, Peru, and formal ethical approval was not required owing to the nature of the study.

## Results

### Room ventilation

38 CO_2_ tracer gas experiments were performed in 6 study rooms. Ventilation increased in each room as a result of the intervention in all experiments (*p* < 0.0001; Wilcoxon signed-ranks test; Fig. 1). Median ventilation was 5.3 ACH pre-intervention and 16 ACH post-intervention. In the general medical out-patients waiting room with the new skylight, ventilation increased from mean 5.5 to 14.5 ACH. In the adjacent medical consulting room, ventilation increased from mean 4.6 to 17.4 ACH. In the X-ray department waiting room ventilation increased from mean 10.5 to 15.6 ACH. In the respiratory medicine out-patients & TB clinic waiting room ventilation increased from mean 8.0 to 15.3 ACH. In the respiratory medicine out-patients consulting room ventilation increased from mean 2.7 to 11.9 ACH. In the general medical and respiratory medicine out-patients waiting room mean ventilation was 8.1 ACH, and in the new purpose built respiratory out-patients waiting room, mean ventilation was 61.6.

### Risk of TB infection

The following data were collected by direct observation and review of clinic records and were entered into the airborne infection model for number of susceptibles (S), and number of infectors (I). In the general medical out-patients waiting room (Room 1) there were on average 385 people (patients and those accompanying them) at any one time, and 1 untreated smear positive TB case was attended to daily. Similarly, at any one time there were 65 people in the X-ray waiting corridor (Room 3) and 0.5 TB cases attended daily; 14 people in the respiratory/TB clinic waiting area (Room 4) and 10 TB cases attended daily; 120 people in the shared out-patients waiting room (Room 6), with 0.3 infectious TB cases attended daily; and 14 people in the newly built waiting room. Waiting time was considered to be 3 h after conferring with staff, hence t = 3 h was used arbitrarily in the model. In the two consulting rooms (Rooms 2 and 5), 2 healthcare workers (a consulting doctor and one nurse) were considered to be at risk for a total of 6 h/day (representing a morning and afternoon clinic), exposed to one patient at a time.

**Fig. 2:**
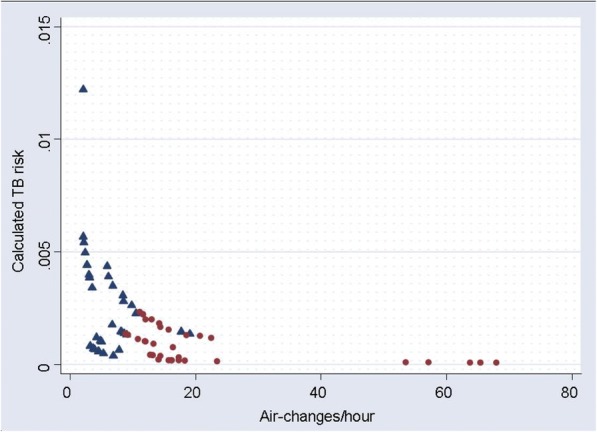
Risk of TB infection and room ventilation. The risk of TB infection for patients waiting an average of 3 h in a waiting room, or for a healthcare worker working for 6 h in a consulting room, is shown. TB risk was calculated using the room ventilation rate measured in each room either pre-intervention (blue triangles) or post- intervention to improve natural ventilation (red circles)

The calculated risk of TB infection for patients waiting 3 h in waiting rooms, or for healthcare workers working 6 h in the consulting rooms is shown in Fig. 2, for room ventilation as measured pre- and post-intervention. The calculated risk of TB transmission decreased in each room as a result of the intervention increasing room ventilation in all experiments (*p* < 0.0001; Wilcoxon signed-ranks test). The median reduction in risk of TB infection was 72% (inter-quartile range 51–82%).

## Discussion

This study has demonstrated that simple, low cost modifications to existing infrastructure can greatly improve natural ventilation in healthcare settings, in this case in hospital consulting rooms and waiting rooms. Such rooms are likely to contain infectious TB patients and the increased ventilation considerably reduced TB transmission risk to staff and other patients calculated using a standard airborne infection model. These interventions to improve natural ventilation within existing infrastructure were achieved at minimal or modest cost. These findings demonstrate the potential for simple implementation of effective environmental control measures for preventing TB transmission in healthcare settings, which is of particular importance in the context of the roll out of HIV care.

A number of guidelines exist for preventing TB transmission in healthcare facilities, including WHO guidance for low resource settings [13, 14]. TB infection control involves administrative measures to ensure the prompt diagnosis, isolation and initiation of effective treatment of TB patients; environmental control measures to reduce the airborne concentration of infectious droplets; and personal respiratory protection. TB infection control has often been neglected, especially in low resource settings, and is frequently limited to small areas of healthcare facilities perceived to be at highest risk, such as respiratory isolation rooms. However, it is TB patients who are untreated (prior to diagnosis) or inadequately treated (due to delayed diagnosis of drug-resistant TB) who are likely to be the most infectious [8, 15, 18, 19]. These types of patients are likely to be found in waiting rooms, out-patient clinics, X-ray departments, and emergency rooms. In a Canadian study, 47% of 250 TB patients made 258 visits to the emergency department prior to diagnosis, and 95% of source case nosocomial infectiousness time occurred in the emergency department [20]. In this study, new TB patients were documented in overcrowded waiting rooms, where average waiting times were around 3 h. It is likely that these poorly ventilated, overcrowded areas are responsible for considerable TB transmission, as suggested by the airborne infection model.

Administrative control measures such as triage of coughing patients or use of screening algorithms in emergency departments are undoubtedly useful, but their effectiveness is limited [21, 22]. In a study from Peru, up to 31% of TB patients attending an emergency department had entirely unsuspected TB, and would not have been identified by screening protocols [23]. A modelling study of the emergence of extensively drug-resistant (XDR) TB in KwazuluNatal predicted that administrative control measures alone would avert just 10% of new XDR-TB cases, compared with 33% if natural ventilation were implemented [24]. Thus environmental control measures such as room ventilation or upper-room ultraviolet light [25] that protect against both the expected and the unexpected TB case across healthcare facilities therefore assume increased importance. Mechanical ventilation is expensive, and requires specific expertise in design, installation, and maintenance. Poor maintenance of mechanical ventilation, which may lead to serious adverse consequences such as positive instead of negative pressure, has been widely documented in developed countries, and associated with nosocomial TB outbreaks [26–31]. Maintenance of such systems is even harder in low resource settings, where budget and parts for annual maintenance may be unavailable. Furthermore, high air exchange mechanical ventilation is limited by its cost to high-risk areas such as respiratory isolation rooms. Areas such as waiting rooms or consulting rooms are often ventilated at much lower rates, reflecting odour and heat considerations rather than control of airborne infection. In contrast to mechanical ventilation, natural ventilation, where climate permits, is applicable across most parts of healthcare facilities, including large, overcrowded waiting rooms such as those in this study [12]. It is difficult to provide consistent directional airflow with natural ventilation, however where there are prevailing winds (as is the case in this study in Lima which is a coastal city) seating arrangement may be adjusted for additional safety. In the two consulting rooms in this study, for example, the chairs for the consulting doctors were located adjacent to the windows open to prevailing breezes, with patients being consulted ‘downwind’ across a desk.

This study has shown that even simple modifications to existing infrastructure can facilitate high rates of natural ventilation. This is important because in many healthcare facilities resources may not be available for new buildings, or logistical difficulties may hinder relocation of a particular hospital service to a more appropriate site. Much higher rates of ventilation may be achieved when infrastructure is specifically designed to facilitate natural ventilation, such as the new respiratory out-patients waiting room in this study, where 54–68 ACH were measured. It is therefore important that TB infection control is prioritized at the design stage when planning new healthcare facilities, allowing optimization of environmental control measures such as natural ventilation, with carefully planned layout of services and direction of patient flow to minimise time spent in overcrowded areas by potentially infectious patients. For example, the siting of a waiting area outside where climate permits is likely to have a beneficial effect on airborne disease transmission control [32]. These architectural design and patient flow considerations are especially important in the roll out of HIV care, where highly susceptible persons are required to spend often long periods in very overcrowded facilities, sharing airspace with those who are likely to have active TB disease. There exists considerable potential for funding agencies to require that TB infection control be an integral part of TB and HIV control initiatives where new building or remodelling of existing infrastructure is planned. This need is of great urgency when considering the number of new healthcare facilities already under construction around the world as HIV treatment is scaled-up.

Limitations of this study include the assumptions inherent in the airborne infection model, and lack of precision in estimating the prevalence of new TB cases in waiting and consulting rooms owing to the small amount of data collected. However, we believe that the estimates of TB risk are conservative, because only new TB cases were considered to be infectious, and no allowance was made for the possibility of drug resistant TB, where those with undiagnosed drug resistant TB being treated with first line therapy would still be infectious [8]. However, it is the reduction in TB risk that is the main outcome measure of interest in this study. When the only variable changing in the model pre- and post- intervention is room ventilation, the absolute numbers of infectors, or the exact time spent in the waiting room, have almost no influence on the percentage risk reduction in TB transmission observed. Another limitation is that relatively small numbers of ventilation measurements were made in each room, but this is mitigated by the magnitude of the increases in ventilation following the intervention observed consistently in each room. The carbon dioxide tracer gas technique has limitations, especially in not being able to detect variations in ventilation rates around a room if only one sensor is used [33], and through the fact that carbon dioxide is produced by room occupants, in contrast to other tracer gases such as SF_6_. However carbon dioxide use has the advantages of low cost, practicality, and safety considerations for occupied buildings. Most importantly, any limitations in the technique used could be assumed to apply equally to the pre- and post-intervention measurements, minimising impact on the outcome of interest, the reduction of TB transmission risk.

## Conclusions

This study has demonstrated that high rates of natural ventilation may be achieved in healthcare facilities through simple modifications to existing infrastructure, greatly reducing the risk of TB infection for little or no cost. Where climate permits, such architectural modifications have great potential for reducing TB transmission in healthcare facilities and other institutional settings, and are ideally suited to low resource settings. In the current era of dual HIV and TB epidemics, and emerging MDR and XDR-TB, these simple interventions may help to prevent healthcare settings from propagating the very diseases they are attempting to treat.

## Abbreviations

ACH: Air Changes per hour
CO_2_: Carbon dioxide
HIV: Human Immunodeficiency Virus
TB: Tuberculosis
WHO: World Health Organisation
XDR-TB: Extensively drug-resistant tuberculosis
